# BCG as a Vector for Novel Recombinant Vaccines against Infectious Diseases and Cancers

**DOI:** 10.3390/vaccines8040736

**Published:** 2020-12-04

**Authors:** Abu Salim Mustafa

**Affiliations:** Department of Microbiology, Faculty of Medicine, Kuwait University, Safat 13110, Kuwait; abu.mustafa@ku.edu.kw

Bacillus Calmette–Guérin (BCG) has been widely used globally as a prophylactic vaccine to protect against tuberculosis (TB) for about a century. BCG is an attenuated strain of a pathogenic strain of *Mycobacterium bovis*, which was isolated from the milk of a cow suffering from TB mastitis. To attenuate *M. bovis*, it was sub-cultured by Albert Calmette and Camille Guérin for 13 years and passaged for 213 times on an artificial medium in vitro. The resulting organism lost its pathogeneses for animals and was termed as BCG. Interestingly, the attenuated *M. bovis* BCG retained its immunogenic characteristics and protected vaccinated cows against challenge with pathogenic *M. bovis* [1]. These results established the safety and efficacy of BCG vaccine in animals.

In 1921, BCG vaccination was used for the first time in an infant who was a close contact of a TB patient. On the follow-up, there were no serious side effects, and the child did not develop any signs of TB disease. Based on this success, additional newborns were vaccinated over several years and no ill effects were reported [1]. Thus, for the first time, a safe and apparently effective vaccine was available for protection against TB in humans. It is estimated that >4 billion people have been vaccinated with BCG in the world, and >120 million doses of BCG are administered annually, which makes it the most widely used vaccine in humans [2].

The protection against TB and other mycobacterial diseases in people vaccinated with BCG is due to the presence of mycobacterial antigens that activate protective immune responses against mycobacterial diseases like TB and leprosy [3,4,5]. However, BCG has also shown protection against diseases of non-mycobacterial origin due to its non-specific immune activator characteristics [4]. Therefore, BCG has been used as a vector for the expression and delivery of both mycobacterial and non-mycobacterial antigens. The recombinant (r)BCGs thus generated have been used as novel recombinant vaccines for protection against both infectious and non-infectious diseases.

rBCG strains expressing *M. tuberculosis*-specific antigens have been produced and evaluated in the induction of immune responses to the expressed antigens and protection against TB in experimental animals [6,7,8]. The experiments with rBCG have been further advanced to conduct phase 1 clinical trials in humans. The results showed that a rBCG strain, GamTBvac, was safe in volunteers previously vaccinated with BCG, and induced immune responses to *M. tuberculosis*-specific antigens [9]. Phase 2 clinical trials with GamTBvac are in progress [10].

Several rBCG strains expressing HIV antigens have been developed and assessed for the induction of specific T-cell responses in animal models [11]. A rBCG strain expressing the HIV Gag (BCG[pWB206]) induced 16-fold greater Gag-specific CD8^+^ T cells than mice immunized with recombinant modified vaccinia virus Ankara (MVA)-Gag. In addition, mice vaccinated with BCG[pWB206] were protected from a surrogate vaccinia virus challenge [12]. In mice, rBCG-pMyong2-p24 elicited enhanced p24-specific immune responses as shown by increased proliferation of HIV-1 Gag-specific CD4^+^ and CD8^+^ T cells, gamma interferon and antibody production, and cytotoxic T lymphocytes responses [13].

rBCG strains expressing HIV antigens have also been tested in prime-boost models [12,14,15]. Levels of Gag-specific CD4 T cells were approximately 5 fold higher in mice primed with BCG[pWB206] and boosted with MVA-Gag than in those receiving the MVA-Gag boost alone [12]. rBCG expressing the HIVACAT T-cell immunogen (HTI), when delivered in combination with chimpanzee adenovirus (ChAd)Ox1.HTI in mice, induced HIV-1-specific T-cell responses [15]. Furthermore, priming with rBCG doubled the magnitude of the T-cell response in comparison with ChAdOx1.HTI alone while maintaining its breadth [15].

Several recently published reports have suggested the protective effect of BCG vaccine against the development of corona virus disease (COVID)-19 and death [16,17,18]. However, other reports do not support this suggestion [19,20]. It is quite unlikely that BCG by itself can provide substantial protection against SARS-CoV-2 infection solely due to its activation of innate immunity. However, rBCG strains expressing spike protein of SARS-CoV-2 may provide a high degree of protection against COVID-19 due to the activation of both innate and virus-specific adaptive immune responses [21]. The high level of safety records of BCG in healthy humans, its potent adjuvant activity, non-requirement of a cold chain and low cost for manufacturing the vaccine makes rBCG an interesting candidate for protection against COVID-19 [21].

Mice immunized with a rBCG expressing gp63 antigen of Leishmania were protected against challenges with Leishmania promastigotes [22] and *Leishmania major* [23]. Similarly, immunization of mice with a rBCG strain expressing four antigens of *Trypanosoma cruzi* resulted into the induction of parasite antigen-specific T helper (Th1) and Th17 cytokine responses and provided significant protection against challenge with the parasites with a low degree of cardiac lesions 120 days after infection [24].

A host of cancers have been targeted for immunotherapy using rBCG [25]. VPM1002BC is a live rBCG prepared by insertion of listerolysin gene from *Listeria monocytogenes* into the *urease c* gene of BCG. The intravesical application of VPM1002BC in patients with non-muscle invasive bladder cancer was found safe and well tolerated by patients and resulted in the induction of Th1-type immune response [26].

A rBCG strain expressing the detoxified S1 pertussis toxin (rBCG-S1PT) proved more effective than wildtype BCG (WT-BCG) in increasing survival time in an experimental mouse model of bladder cancer. Furthermore, human peripheral blood leukocytes stimulated with rBCG-S1PT produced increased levels of IL-6, IL-8, and IL-10; enhanced the expression of CD25 and CD69 on human CD4^+^ T cells; and induced higher cytotoxicity to MB49 bladder cancer cells than WT-BCG [27].

Two rBCG strains expressing Streptococcal inhibitor of complement (Sic) [rBCG-Sic], and d-alanyl carrier protein ligase (dltA) [rBCG-Sic] were tested in a growth inhibition assay using two bladder cancer cell lines (5637, T24). The growth inhibitory effects of rBCGs on bladder cancer cells were significantly enhanced as compared to WT-BCG. After 8 h of infection, the levels of internalization were higher in rBCG-infected bladder cancer cells than in BCG-infected cells, and cells infected with rBCGs showed increased release of antitumor cytokines, such as IL-6/12, TNF-α, and INF-γ, resulting in inhibition of bacterial killing and immune modulation via antimicrobial peptides [28].

The immunization of mice with a rBCG strain, expressing human granulocyte macrophage colony stimulating factor (hGM-CSF) gene and the Epstein–Barr virus gene BZLF1, induced antigen-specific antibodies. The specific cytotoxic effects of the spleen cells from the rBCG group on Epstein–Barr virus-positive tumor cells were significantly higher than the cytotoxic effects of the control group cells. Morphological observations of the tumor sections from the rBCG-immunized mice showed the infiltration of CD4^+^ T and CD8^+^ T lymphocytes into the tumor tissues. The average tumor volume in the rBCG group was less than the average tumor volume in the control group [29].

rBCG strains expressing single mycobacterial protein antigens have shown efficacy in enhancing cytotoxicity on superficial bladder cancers in vitro and orthotopic murine bladder models [29]. Furthermore, rBCG strains expressing multiple mycobacterial antigens induced predominantly Th1-type immune responses, inhibited tumor growth, and prolonged the survival of mice with bladder tumor [29]. Several rBCG strains have been constructed that secrete cytokines with anti-tumor activity, e.g., IL-2, Il-12 IL-18 and IFN-α. IL-2-based rBCG strains have shown that, relative to BCG alone, IL-2 secreting rBCG strains can improve antigen-specific proliferation, induce a more favorable IFN-γ:IL-4 ratio, elicit higher levels of Th1 cytokines, and enhance antitumor cytotoxicity [30]. The IL-18-secreting rBCG strain induced increased secretion of IFN-γ and GM-CSF, decreased production of IL-10, increased cellular proliferation, induced higher production of IFN-γ-secreting cells in mouse splenocytes and enhanced BCG-induced macrophage cytotoxicity against murine bladder tumor cells in a dose-dependent manner [31]. IFN-α-secreting rBCG strains were more effective than WT-BCG in inducing IFN-γ production from peripheral blood mononuclear cells (PBMC) and inducing PBMC cytotoxicity against bladder cancer cell lines in vitro [32].
